# Horizontal Gene Transfer Contributes to Plant Evolution: The Case of *Agrobacterium* T-DNAs

**DOI:** 10.3389/fpls.2017.02015

**Published:** 2017-11-24

**Authors:** Dora G. Quispe-Huamanquispe, Godelieve Gheysen, Jan F. Kreuze

**Affiliations:** ^1^Department of Molecular Biotechnology, Ghent University, Ghent, Belgium; ^2^International Potato Center (CIP), Lima, Peru

**Keywords:** HGT (horizontal gene transfer), *Agrobacterium*, Ipomoea batatas (L.) Lam., T-DNAs, evolution

## Abstract

Horizontal gene transfer (HGT) can be defined as the acquisition of genetic material from another organism without being its offspring. HGT is common in the microbial world including archaea and bacteria, where HGT mechanisms are widely understood and recognized as an important force in evolution. In eukaryotes, HGT now appears to occur more frequently than originally thought. Many studies are currently detecting novel HGT events among distinct lineages using next-generation sequencing. Most examples to date include gene transfers from bacterial donors to recipient organisms including fungi, plants, and animals. In plants, one well-studied example of HGT is the transfer of the tumor-inducing genes (T-DNAs) from some *Agrobacterium* species into their host plant genomes. Evidence of T-DNAs from *Agrobacterium* spp. into plant genomes, and their subsequent maintenance in the germline, has been reported in *Nicotiana*, *Linaria* and, more recently, in *Ipomoea* species. The transferred genes do not produce the usual disease phenotype, and appear to have a role in evolution of these plants. In this paper, we review previous reported cases of HGT from *Agrobacterium*, including the transfer of T-DNA regions from *Agrobacterium* spp. to the sweetpotato [*Ipomoea batatas* (L.) Lam.] genome which is, to date, the sole documented example of a naturally-occurring incidence of HGT from *Agrobacterium* to a domesticated crop plant. We also discuss the possible evolutionary impact of T-DNA acquisition on plants.

## Introduction

Horizontal gene transfer (HGT) can be defined as the acquisition of genetic material from another organism without being its offspring. It contrasts with vertical gene transfer, which is the acquisition of genetic material from an ancestor. HGT is a universal phenomenon and occurs frequently among prokaryotes. Bacteria have acquired a variety of important traits including antibiotic resistance, pathogenesis and metabolic pathways, via HGT. These horizontal gene acquisitions enabled bacteria to explore new habitats and hence facilitated their rapid evolution (Maiden, 1998; Ochman et al., 2000; Gogarten et al., 2002; Hotopp et al., 2007). In contrast to its rather common occurrence in prokaryotes, examples of HGT in eukaryotes have been reported only infrequently. However, that appears to be changing as recent discoveries indicate the possible contribution of HGT to the acquisition of traits with adaptive significance, suggesting that HGT is an important driving force in the evolution of eukaryotes, as well as prokaryotes. In this paper, we review HGT in higher organisms emphasizing examples involving *Agrobacterium* species and plants. We also discuss the possible evolutionary impact of the transferred genes on their respective hosts.

## HGT in Eukaryotes

Horizontal gene transfer played a pivotal role in the origin of eukaryotes. Endosymbiosis and the subsequent genetic integration of entire organisms gave rise to the mitochondria and plastids (Talianova and Janousek, 2011). Advances in sequencing technologies in combination with ever increasing amounts of sequence data have facilitated the identification of additional examples of HGT in eukaryotes. In most instances, these were identified by chance while sequencing for other purposes or as a result of phylogenetic incongruences while attempting to establish evolutionary relationships. Examples include DNA transfer from bacteria, fungi and plants to bdelloid rotifers (Gladyshev et al., 2008), bacteria to insects (Hotopp et al., 2007), bacteria and fungi to nematodes (Noon and Baum, 2016), fish to fish (Graham et al., 2008), bryophytes to ferns (Li et al., 2014), and bacteria to plants (White et al., 1983; Matveeva and Lutova, 2014; Kyndt et al., 2015).

A particularly interesting example of HGT is the transfer of fungal genes to the pea aphid (*Acyrthosiphon pisum*) (Moran and Jarvik, 2010). Genes coding for carotenoid synthase/cyclase and carotenoid desaturase enzymes had not been reported in animals until their discovery in the pea aphid genome. Carotenoid biosynthesis genes are responsible for the body pigmentation in pea aphids. Body color is considered an ecologically important trait that influences the susceptibility of pea aphids to predators and/or parasites. Ladybugs (Coccinellidae) prefer to attack red aphids while parasitic wasps are more likely to lay their eggs in green aphids. Phylogenetic analysis of these genes in the pea aphid (Nováková and Moran, 2012) indicated that they had been obtained from fungi. Further analyses suggested that these genes were acquired early in the radiation of this group (Nováková and Moran, 2012).

Despite being one of the oldest groups of land plants, the majority of living ferns resulted from a relatively recent diversification following the arrival of angiosperms. In order to exploit the new understory habitats created by angiosperm dominated ecosystems, ferns evolved strategies to thrive under the low light conditions created by the angiosperm canopy. In adapting to these conditions, ferns acquired an unconventional chimeric photoreceptor, called neochrome, that fuses red sensing phytochrome and blue sensing phototropin modules into a single gene, thereby optimizing phototropic responses (Li et al., 2014). The recent analysis of 434 transcriptomes and 40 genomes of plants and algae demonstrated that ferns acquired this gene from hornworts (a bryophyte lineage) via HGT about 179 million year ago (Li et al., 2014).

Horizontal gene transfer seems to have played an important role also in the transition from the aquatic to the terrestrial environment. A novel genome analyses of the moss *Physcomitrella patens* reveal that 57 families of nuclear genes were acquired by HGT from prokaryotes, fungi or viruses. These genes have strong implications on plant-specific activities, such as xylem formation, plant defense, and hormone biosynthesis. The study suggests that many of these genes were transferred to the ancestors of green or land plants (Yue et al., 2012). Other examples of HGT in plants involve the case of parasitic plants (Yang et al., 2016). The transcriptome analyses of three parasitic members of Orobanchaceae family show the occurrence of 52 high-confidence HGT events. Genes acquired by HGT are preferentially expressed in the haustorium, the host connecting organ of parasitic plants, proposing that these genes are contributing to the unique adaptive feeding structure of parasitic plants.

## HGT from *Agrobacterium* Species to Plants (The Classical Model)

*Agrobacterium*-mediated plant genetic transformation is probably the best studied and best understood system of transkingdom gene transfer. *Agrobacterium* is a plant pathogenic bacterium that causes neoplastic growth, i.e., uncontrolled cell division in host plants resulting in crown galls or in proliferating roots following the transfer of a segment of its DNA into the host cell genome.

Most of the bacterial genes necessary for the DNA transfer are located in a large tumor- or root-inducing plasmid (Ti/Ri plasmid) which also contains that part of the plasmid that is transferred (T-DNA). During *Agrobacterium* infection, plant-derived phenolics trigger the expression of the bacterium’s virulence genes, and the encoded proteins subsequently mediate the T-DNA transfer to the host plant cell. The final destiny of the T-DNA in the host cell is dependent on various interactions between *Agrobacterium* and plant proteins. Several host cell pathways are utilized to ensure that the T-DNA is imported to the nucleus and integrated into the host genome (Lacroix and Citovsky, 2016). Expression of the T-DNA genes in the plant can alter the physiology to stimulate cell division and root growth. *IaaM* and *iaaH* encode enzymes for the biosynthesis of auxin that is essential for crown gall development (Zhang et al., 2015). Several *rol* (root loci) genes are involved in root formation while the function of several T-DNA genes such as *C-prot* is still unknown (Otten et al., 1999). Opines are also encoded on the T-DNAs, they are utilized as carbon and nitrogen sources by invading bacteria and their presence can alter the biological root environment, particularly, root associated bacterial populations (Oger et al., 1997). *Acs* encodes the key enzyme for the biosynthesis of the opine called *agrocinopine* while *mis* is a mikimopine synthase and *mas* a mannopine synthase.

**FIGURE 1:**
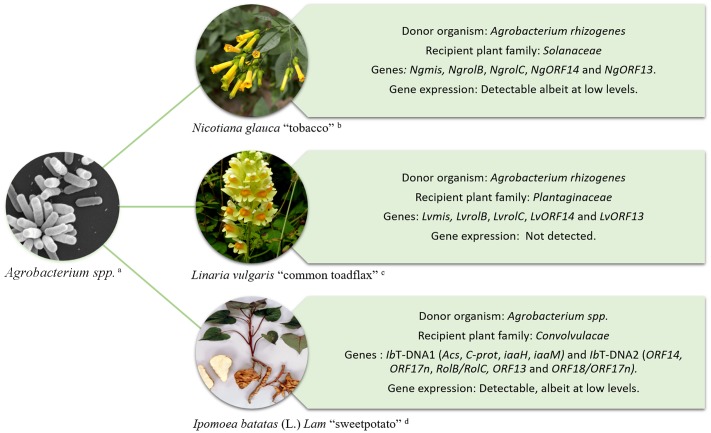
Schematic representation of HGT from *Agrobacterium* to plants. Examples include; *Nicotiana glauca* “tobacco” (White et al., 1983) – upper right, *Linaria vulgaris* “common toadflax” (Matveeva et al., 2012) – middle right, and *Ipomoea batatas* (L.) *Lam* “sweet potato” (Kyndt et al., 2015) – lower right. Imagens courtesy of (a) Jing Xu, Indiana University, (b) A. H. Harris, Texas University at El Paso, (c) Graham Calow, NatureSpot (http://www.naturespot.org.uk/species/common-toadflax), and (d) International Potato Center (CIP).

The ability of *Agrobacterium* to transform plants has been exploited for decades as a means to introduce foreign genes of interest into crop plants (Tzfira and Citovsky, 2006; Gelvin, 2009). However, *Agrobacterium*-mediated HGT is not restricted to the production of genetically modified crops. Evidence of the naturally occurring transfer of T-DNA genes from *Agrobacterium* into plant genomes and their subsequent maintenance in the germline has been documented in *Nicotiana*, *Linaria*, and more recently *Ipomoea* species **Figure 1** (White et al., 1983; Intrieri and Buiatti, 2001; Matveeva et al., 2012; Pavlova et al., 2014; Kyndt et al., 2015). In these examples, the transferred genes are fixed and are expressed in the host plant’s lineage suggesting that they might have a functional role.

## HGT from *Agrobacterium rhizogenes* to *Nicotiana* and *Linaria*

More than three decades ago White et al. (1983) detected a region in the genome of *Nicotiana glauca* homologous to regions in the T-DNA of the Ri plasmid of *Agrobacterium rhizogenes*. The region was called cellular T-DNA (cT-DNA) (White et al., 1983). The cT-DNA in the *N. glauca* genome was initially described as an imperfect inverted repeat that contained two homologs to *rol* genes, *NgrolB* and *NgrolC* (Ng, *N. glauca*). Later, the cT-DNA was found to contain two additional genes corresponding to open reading frames *ORF13* and *ORF14* (Aoki et al., 1994). The discovery of mikimopine synthase (*mis*) sequences (*NgmisL* and *NgmisR*) in the *N. glauca* cT-DNA indicated that it originated from a mikimopine-type Ri plasmid (Suzuki et al., 2002).

PCR analysis and southern hybridization confirmed the acquisition of cT-DNA by *N. glauca* (Furner et al., 1986). Intrieri and Buiatti (2001) screened a total of 42 *Nicotiana* species for the presence of *rolB, rolC, ORF13* and *ORF14*, and at least one of those genes was detected in the genome of 15 species. Phylogenetic analyses concluded that the *rol* genes seemed to follow the evolution of the genus *Nicotiana*. This study (Intrieri and Buiatti, 2001) also suggested that more than one independent infection of *Nicotiana* by *A. rhizogenes* occurred in ancient times. This hypothesis was recently corroborated through deep sequencing of the genome of the ancestral tobacco species *Nicotiana tomentosiformis* (Chen et al., 2014). The genome of *N. tomentosiformis* contains four cT-DNAs all of which are derived from different *Agrobacterium* strains. These cT-DNAs, TA, TB, TC, and TD, each contain an incomplete inverted-repeat structure. The TB region contains an intact mannopine synthase 2′ gene (TB- *mas2*′) that is highly expressed in roots of some *N. tabacum* cultivars. These results suggest that the TB-*mas2*′ gene could have been selected in some tobacco populations by nature or by tobacco growers, as a result of changes in the root metabolism of these plants (Chen et al., 2016).

cT-DNA sequences are not restricted to the genus *Nicotiana*. Indeed, they have also been found in species belonging to the genus *Linaria*, primarily within sections *Linaria* and *Speciosae* (Pavlova et al., 2014). Two copies of cT-DNA are present in *Linaria vulgaris* and are imperfect direct repeats. The *Linaria* cT-DNA appears to have originated from an ancient infection by a mikimopine strain of *A. rhizogenes*. Among the cT-DNA genes, *rolC* is the most conserved gene in the *Linaria* group and it contains an intact ORF. However, reverse transcriptional (RT) real-time PCR assays carried out using *L. vulgaris* internodes, leaves and roots under *in vitro* conditions have shown that *rolC* and the other cT-DNA genes are not expressed in these tissues (Matveeva et al., 2012).

## HGT from *Agrobacterium* to *Ipomoea* spp.

Sweet potato [*Ipomoea batatas* (L.) Lam.] belongs to genus *Ipomoea*. *Ipomoea* is the largest genus in the family Convolvulaceae and contains 600–700 species. Over half of *Ipomoea* spp. are concentrated in the Americas, where they are distributed as cultigens, medicinal plants and weeds (Huaman, 1992). Series Batatas is a small group of taxa within the genus *Ipomoea* that contains 13 species that are considered to be closely related to sweet potato (Nimmakayala et al., 2011). Members of this series include *Ipomoea cordatotriloba*, *I. cynanchifolia*, *I. grandiflora*, *I. lacunosa*, *I. leucantha*, *I. littoralis*, *I. ramosissima*, *I. umbraticola*, *I. tabascana*, *I. tenuissima*, *I. tiliacea*, *I. trifida*, and *I. triloba*. The basic chromosome number of the series is 15 whereas the cultivated sweet potato is a hexaploid species (2n = 6x = 90). However, tetraploid (2n = 4x = 60) variants of *I. batatas* have also been reported (Bohac et al., 1993; Roullier et al., 2013) and these are sometimes referred to as tetraploid *I. trifida* or wild sweet potatoes in the scientific literature. Today, sweet potato is a staple food crop in many areas of the world. However, its botanical origins and the details concerning its domestication remain obscure.

The discovery of *Agrobacterium* genes *Ib*T-DNA1 and *Ib*T-DNA2 in the sweet potato genome represents the only known example of an ancient HGT that occurred in, what is today, a domesticated crop (Kyndt et al., 2015). Both regions, *Ib*T-DNA1 and *Ib*T-DNA2 were fortuitously detected during an analysis of small interfering RNA (*siRNA*) in the sweet potato cultivar Huachano. Plants of cv. Huachano contain an *Ib*T-DNA1 with at least 4 ORFs with significant homology to the bacterial genes tryptophan-2-monooxygenase (*iaaM*), indole-3-acetamide hydrolase (*iaaH*), C-protein (*C-prot*) and agrocinopine synthase (*Acs*) and an *Ib*T-DNA2 containing at least five ORFs with significant homology to *ORF14, ORF17n, RolB/RolC, ORF13*, and *ORF18/ORF17n*. The insertion of *Ib*T-DNA1 has been corroborated by sequence analysis of a bacterial artificial chromosome (BAC) clone of sweet potato cv. Xu781. The BAC sequence revealed that the complete *Ib*T-DNA1 encompassed 21,564 bp and consisted of an inverted repeat. *Ib*T-DNA1 and 2 are transmitted from parent to progeny and the genes are expressed at detectable levels in different sweet potato tissues suggesting that they may have a function (Kyndt et al., 2015).

## The Evolutionary Impact of the Acquisition of T-DNAs In Plants

In general, for any foreign gene to be acquired by a host and stably inherited by its offspring (i) it must enter a cell and be integrated into the recipient genome, (ii) the DNA should not be lost after genome rearrangements during subsequent cell divisions, (iii) the transformed cell must enter the germ line, and finally (iv) the integrated sequence must be preserved in the course of evolution, which is most likely to happen if the gene confers a selective advantage to the recipient organism (Huang, 2013; Lacroix and Citovsky, 2016). In the case of T-DNA genes one could assume another specific requirement which is that the inserted genes must somehow be modified or controlled from their ‘natural’ expression pattern to avoid vigorous cell growth that would be detrimental to survival of the plant. The gold standard to determine gene function resulting from HGT is the existence of a phenotype that is correlated with the presence of those genes. However, changes to the phenotype are not always so obvious and may in fact be difficult to detect.

The phenotypic effect of the *Agrobacterium rol* genes present in *Nicotiana* and *I. batatas* is likely associated with root traits (Matveeva et al., 2012; Kyndt et al., 2015). The suite of genes *rolA, rolB*, and *rolC* when transformed in tobacco plants, is able to induce the full “hairy root syndrome” (Maurel et al., 1991). *RolA* and *rolB* are mutated in *N. tabacum*, while *rolC* is intact. Transgenic tobacco plants bearing only *rolC* display phenotypic changes such as reduced apical dominance, dwarfism, shortened internodes, lanceolate leaves, and early flowering. They also exhibit increased root production when compared to untransformed plants (Shoja, 2010). The exact role of *Ib*T-DNA genes remains to be elucidated, although is known that the larger part of *Ib*T-DNA1 and *Ib*T-DNA2 genes are intact and are expressed.

How plants have avoided the *Agrobacterium* programmed expression of T-DNA sequences after insertion into their genomes to avoid *Agrobacterium* programmed cell proliferation is not clear yet, but several options can be considered. The T-DNA may have integrated in a region of the genome that is transcriptionally inactive, a property which is subsequently imparted on the inserted T-DNA. On the other hand, in sweetpotato the *Ib*T-DNAs were originally discovered by small RNA sequencing and assembly, indicating that the genes -arranged in an inverted repeat- are targeted by the RNA silencing mechanism of the plant and in that way suppressed in their expression, even if they integrated in a transcriptionally active region of the genome (Kyndt et al., 2015). In the case of TB-*mas2′*, evidence suggests that originally it was a functional gene but has lost its expression in *N. tomentosiformis*, perhaps due to gene silencing; whereas it is active in *N. tabacum* (Chen et al., 2016).

The production of storage roots and the ability to easily propagate via rooted vine cuttings are major traits associated with the domestication of the sweet potato. Considering that *Ib*T-DNAs appear to be inherited from wild relatives and some *Ib*T-DNA genes have the potential to change plant physiology (auxin biosynthesis or sensitivity), it is tempting to speculate that *Ib*T-DNAs have conferred an adaptive advantage to the host. A possible association between *rolB/rolC* genes and root parameters (total root yield, dry matter content, and harvest index) was evaluated in a population segregating for *Ib*T-DNA2. No association between the occurrence of these genes and the noted root characteristics was detected, except for root yield at one location (Kyndt et al., 2015). Further study is required to establish the role, if any, of *Ib*T-DNA2 genes in root development. To this end, a functional analysis using CRISPR-Cas9 to knockout single genes, combinations of genes, or the whole *Ib*T-DNA1 and/or 2, would result in plants that could be analyzed in detail for their phenotype and developmental characteristics.

Knowledge of the timing of the ancestral infection as well as details about the infection process (such as whether it occurred as a single event or as multiple independent events), could shed light on the evolutionary impact of the cT-DNA and the *Ib*T-DNA sequences on *Nicotiana* and *Ipomoea* spp. In *Nicotiana*, the data suggest that *Agrobacterium* spp. infected this group multiple times, independently. Incongruences during the phylogenetic analyses of *rol*B in *Nicotiana* were the first evidence of this hypothesis (Intrieri and Buiatti, 2001; Suzuki et al., 2002) which is now gaining general acceptance. Indeed, the four cT-DNAs found in the ancestral tobacco species *N. tomentosiformis* appear to be derived from different *Agrobacterium* strains (Chen et al., 2016). In the sweet potato, *Ib*T-DNA 1 and 2 are present at different loci and segregate independently, i.e., *Ib*T-DNA1 seems to be fixed, while *Ib*T-DNA2 is restricted to only some accessions and segregates at random depending which genes are being analyzed. These differences may reflect different infection events. However, *A. rhizogenes* plasmids typically have two T-DNAs corresponding to *Ib*T-DNA1 and 2 that are transferred independently but often simultaneously.

## Future Perspectives

Investigations about the role of *Agrobacterium* T-DNAs in the evolution of plants are only just beginning. Screening of additional *Ipomoea* species in our labs will demonstrate if T-DNA genes are confined to the cultivated sweet potato, or are also present in some of its wild relatives. The pattern of possible acquisition of *Ib*T-DNAs by other *Ipomoea* species may help to formulate a hypothesis on the role that these sequences have played in the evolution of this crop – and its related species. Although these genes are expressed at detectable levels in sweet potato, and some of them (*rolB*/*rolC*) are associated with root parameters, further analyses are needed in order to clarify their function(s).

## Author Contributions

DQ-H wrote the first draft and JK and GG subsequently contributed to produce the final version.

## Conflict of Interest Statement

The authors declare that the research was conducted in the absence of any commercial or financial relationships that could be construed as a potential conflict of interest.

## References

[B1] Aoki S. Kawaoka A. Sekine M. Ichikawa T. Fujita T. Shinmyo A. (1994). Sequence of the cellular T-DNA in the untransformed genome of *Nicotiana glauca* that is homologous to ORFs 13 and 14 of the Ri plasmid and analysis of its expression in genetic tumors of *N. glauca* x *N. langsdorffii*. *Mol. Gen. Genet.* 243 706–710. 10.1007/BF00279581 8028588

[B2] Bohac J. R. Austin D. F. Jones A. (1993). Discovery of wild tetraploid sweetpotatoes. *Econ. Bot.* 47 193–201. 10.1007/BF02862022

[B3] Chen K. Borne F. D. Julio E. Obszynski J. Pale P. Otten L. (2016). Root-specific expression of opine genes and opine accumulation in some cultivars of the naturally occurring genetically modified organism *Nicotiana tabacum*. *Plant J.* 87 258–269. 10.1111/tpj.13196 27125327

[B4] Chen K. Dorlhac de Borne F. Szegedi E. Otten L. (2014). Deep sequencing of the ancestral tobacco species *Nicotiana tomentosiformis* reveals multiple T-DNA inserts and a complex evolutionary history of natural transformation in the genus *Nicotiana*. *Plant J.* 80 669–682. 10.1111/tpj.12661 25219519

[B5] Furner I. J. Huffman G. A. Amasino R. M. Garfinkel D. J. Gordon M. P. Nester E. W. (1986). An *Agrobacterium* transformation in the evolution of the genus *Nicotiana*. *Nature* 319 422–427. 10.1038/319422a0

[B6] Gelvin S. B. (2009). *Agrobacterium* in the genomics age. *Plant physiol*. 150 1665–1676. 10.1104/pp.109.139873 19439569 PMC2719113

[B7] Gladyshev E. A. Meselson M. Arkhipova I. R. (2008). Massive horizontal gene transfer in bdelloid rotifers. *Science* 320 1210–1213. 10.1126/science.1156407 18511688

[B8] Gogarten J. P. Doolittle W. F. Lawrence J. G. (2002). Prokaryotic evolution in light of gene transfer. *Mol. Biol. Evol.* 19 2226–2238. 10.1093/oxfordjournals.molbev.a004046 12446813

[B9] Graham L. A. Lougheed S. C. Ewart K. V. Davies P. L. (2008). Lateral transfer of a lectin-like antifreeze protein gene in fishes. *PLOS ONE* 3:e2616. 10.1371/journal.pone.0002616 18612417 PMC2440524

[B10] Hotopp J. C. D. Clark M. E. Oliveira D. C. Foster J. M. Fischer P. Torres M. C. M. (2007). Widespread lateral gene transfer from intracellular bacteria to multicellular eukaryotes. *Science* 317 1753–1756. 10.1126/science.1142490 17761848

[B11] Huaman Z. (1992). *Systematic Botany and Morphology of the Sweetpotato Plant.* Lima: International Potato Center (CIP).

[B12] Huang J. (2013). Horizontal gene transfer in eukaryotes: the weak-link model. *Bioessays* 35 868–875. 10.1002/bies.201300007 24037739 PMC4033532

[B13] Intrieri M. C. Buiatti M. (2001). The horizontal transfer of *Agrobacterium rhizogenes* genes and the evolution of the genus *Nicotiana*. *Mol. Phylogenet. Evol.* 20 100–110. 10.1006/mpev.2001.0927 11421651

[B14] Kyndt T. Quispe D. Zhai H. Jarret R. Ghislain M. Liu Q. (2015). The genome of cultivated sweet potato contains *Agrobacterium* T-DNAs with expressed genes: an example of a naturally transgenic food crop. *Proc. Natl. Acad. Sci. U.S.A.* 112 5844–5849. 10.1073/pnas.1419685112 25902487 PMC4426443

[B15] Lacroix B. Citovsky V. (2016). Transfer of DNA from bacteria to eukaryotes. *mBio* 7:e00863-16. 10.1128/mBio.00863-16 27406565 PMC4958254

[B16] Li F. W. Villarreal J. C. Kelly S. Rothfels C. J. Melkonian M. Frangedakis E. (2014). Horizontal transfer of an adaptive chimeric photoreceptor from bryophytes to ferns. *Proc. Natl. Acad. Sci. U.S.A.* 111 6672–6677. 10.1073/pnas.1319929111 24733898 PMC4020063

[B17] Maiden M. C. J. (1998). Horizontal genetic exchange, evolution, and spread of antibiotic resistance in bacteria. *Clin. Infect. Dis.* 27 S12–S20. 10.1086/514917 9710667

[B18] Matveeva T. V. Bogomaz D. I. Pavlova O. A. Nester E. W. Lutova L. A. (2012). Horizontal gene transfer from genus *Agrobacterium* to the plant *Linaria* in nature. *Mol. Plant Microbe Interact.* 25 1542–1551. 10.1094/MPMI-07-12-0169-R 23134518

[B19] Matveeva T. V. Lutova L. A. (2014). Horizontal gene transfer from *Agrobacterium* to plants. *Front. Plant Sci.* 5:326. 10.3389/fpls.2014.00326 25157257 PMC4127661

[B20] Maurel C. Barbier-Brygoo H. Spena A. Tempé J. Guern J. (1991). Single rol genes from the *Agrobacterium rhizogenes* TL-DNA alter some of the cellular responses to auxin in *Nicotiana tabacum*. *Plant Physiol.* 97 212–216. 16668373 10.1104/pp.97.1.212 PMC1080986

[B21] Moran N. A. Jarvik T. (2010). Lateral transfer of genes from fungi underlies carotenoid production in aphids. *Science* 328 624–627. 10.1126/science.1187113 20431015

[B22] Nimmakayala P. Vajja G. Reddy U. K. (2011). “Ipomoea,” in *Wild Crop Relatives: Genomic and Breeding Resources*, ed. Kole C. (Berlin: Springer), 123–132.

[B23] Noon J. B. Baum T. J. (2016). Horizontal gene transfer of acetyltransferases, invertases and chorismate mutases from different bacteria to diverse recipients. *BMC Evol. Biol.* 16:74. 10.1186/s12862-016-0651-y 27068610 PMC4828791

[B24] Nováková E. Moran N. A. (2012). Diversification of genes for carotenoid biosynthesis in aphids following an ancient transfer from a fungus. *Mol. Biol. Evol.* 29 313–323. 10.1093/molbev/msr206 21878683

[B25] Ochman H. Lawrence J. G. Groisman E. A. (2000). Lateral gene transfer and the nature of bacterial innovation. *Nature* 405 299–304. 10.1038/35012500 10830951

[B26] Oger P. Petit A. Dessaux Y. (1997). Genetically engineered plants producing opines alter their biological environment. *Nat. Biotechnol.* 15 369–372. 10.1038/nbt0497-369 9094140

[B27] Otten L. Salomone J.-Y. Helfer A. Schmidt J. Hammann P. De Ruffray P. (1999). Sequence and functional analysis of the left-hand part of the T-region from the nopaline-type Ti plasmid, pTiC58. *Plant Mol. Biol.* 41 765–776. 10.1023/A:1006370207379 10737141

[B28] Pavlova O. Matveeva T. Lutova L. (2014). Genome of *Linaria dalmatica* contains *Agrobacterium rhizogenes RolC* gene homolog. *Russ. J. Genet. Appl. Res.* 4 461–465. 10.1134/S2079059714050116

[B29] Roullier C. Duputié A. Wennekes P. Benoit L. Bringas V. M. F. Rossel G. (2013). Disentangling the origins of cultivated sweet potato (*Ipomoea batatas* (L.) Lam.). *PLOS ONE* 8:e62707. 10.1371/journal.pone.0062707 23723970 PMC3664560

[B30] Shoja H. M. (2010). *Contribution to the Study of the Agrobacterium rhizogenes Plast Genes, RolB and RolC, and Their Homologs in Nicotiana tabacum.* Strasbourg: Universite de Strasbourg.

[B31] Suzuki K. Yamashita I. Tanaka N. (2002). Tobacco plants were transformed by *Agrobacterium rhizogenes* infection during their evolution. *Plant J.* 32 775–787. 10.1046/j.1365-313X.2002.01468.x 12472692

[B32] Talianova M. Janousek B. (2011). What can we learn from tobacco and other Solanaceae about horizontal DNA transfer? *Am. J. Bot*. 98 1231–1242. 10.3732/ajb.1000370 21795732

[B33] Tzfira T. Citovsky V. (2006). *Agrobacterium*-mediated genetic transformation of plants: biology and biotechnology. *Curr. Opin. Biotechnol.* 17 147–154. 10.1016/j.copbio.2006.01.009 16459071

[B34] White F. F. Garfinkel D. J. Huffman G. A. Gordon M. P. Nester E. W. (1983). Sequences homologous to *Agrobacterium rhizogenes* T-DNA in the genomes of uninfected plants. *Nature* 301 348–350. 10.1038/301348a0

[B35] Yang Z. Zhang Y. Wafula E. K. Honaas L. A. Ralph P. E. Jones S. (2016). Horizontal gene transfer is more frequent with increased heterotrophy and contributes to parasite adaptation. *Proc. Natl. Acad. Sci. U.S.A.* 113 E7010–E7019. 10.1073/pnas.1608765113 27791104 PMC5111717

[B36] Yue J. Hu X. Sun H. Yang Y. Huang J. (2012). Widespread impact of horizontal gene transfer on plant colonization of land. *Nat. Commun.* 3 1152. 10.1038/ncomms2148 23093189 PMC3493653

[B37] Zhang Y. Lee C.-W. Wehner N. Imdahl F. Svetlana V. Weiste C. (2015). Regulation of oncogene expression in T-DNA-transformed host plant cells. *PLOS Pathog.* 11:e1004620. 10.1371/journal.ppat.1004620 25615824 PMC4304707

